# Pathology in Its Relations to General Biology1Abstracts from an address delivered at the formal opening of the Biological Laboratory of the University of Toronto, on December 20th, 1889.

**Published:** 1890-03

**Authors:** William H. Welch

**Affiliations:** Pathologist to the Johns Hopkins Hospital and Professor of Pathology, Johns Hopkins University, Baltimore


### CASES, SELECTIONS, TRANSLATIONS.

### PATHOLOGY IN ITS RELATIONS TO GENERAL BIOLOGY.[1]

By William H. Welch, M D.,
*Pathologist to the Johns Hopkins Hospital and Professor of Pathology, Johns Hopkins University, Baltimore.*

Biology in its widest significance is the study of life in all its forms and activities, both normal and abnormal. No branch of human knowledge can exceed this in interest and importance; none has made greater advances during this century of scientific progress; none has achieved greater triumphs for human welfare; none has influenced more profoundly modern philosophical thought.

Pathology is the study of life in its abnormal forms and activities. The relations of pathology to practical medicine are necessarily so essential and intimate that the broader conception of this science as a part of biology is in danger of being lost from view. I deem it, however, important for the scientific status and advancement of pathology to keep in mind and to emphasize its relations to general biology, not less than those to practical medicine.

To-day it is our conviction that these fundamental facts can be discovered in no other way than by observation and experiment. The adoption of this, the only scientific method of investigation, has, with the aid of modern instruments and devices, not only greatly enriched medical science, but it has overthrown the era in which, among scientific physicians, exclusive schools of doctrine can prevail. The scientific physician no more than the scientific chemist can yield adherence to any exclusive dogma. To the one as to the other no way which leads to truth is debarred.

If one seeks an illustration of immediate practical results of the modern investigations of the living germs which cause disease, let him turn his attention to the revolution thereby wrought in surgical procedures. The possibility which is now in the hands of the surgeon of keeping wounds free from all external infection is a boon to humanity not less than the introduction of vaccination.

In the first place, I claim that pathology as a science, quite independently of any practical or useful applications whatever, is as legitimate and worthy an object of pursuit as any of the natural sciences. In and for

[1] Abstracts from an address delivered at the formal opening of the Biological Laboratory of the University of Toronto, on December 20th, 1889.

itself alone it deserves to be studied. Its methods are those of observation and experiment as in other biological sciences. Its subject-matter is any living thing which deviates from the normal condition. It is not less interesting and important to learn the nature and causes of abnormalities in form and function than it is to become familiar with the normal, and when this knowledge may aid in the prevention and relief of suffering added dignity and interest are imparted to the study.

As there comes a line where the distinction between the normal and the abnormal is shadowy and uncertain, so the separation between normal and pathological biology is not sharp. The province of the one encroaches at many points upon that of the other. Mutual aid is to be derived from a closer union between normal and pathological biology. The pathologist should not be content with methods of research less perfect than those employed in normal biology. He should not rest satisfied with results which stop at the mere description and classification of morbid processes. To be able to give a name to some pathological lesion, and to make it fit into some accepted scheme of classification, should not be the sole aim of pathological study. Pathological processes should be studied with the aim of elucidating their real nature, development and causes, their mutual relations and their dependence upon underlying laws. The purely descriptive phase of development of any natural science can be only temporary and unsatisfactory. The more a pathologist is imbued with the spirit of modern biology, the less content will he be to stop at this descriptive phase.

As has already been mentioned, pathology has to do with abnormalities, not in man alone, but in all living things, both animal and vegetable. The points of contact between animal and vegetable pathology are more numerous than might at first glance appear. The student of animal pathology can draw many instructive lessons from such subjects as the behavior of wounds and the parasitic affections in plants.

We are most of us probably inclined to think too much of the separation between the pathology of man and that of the lower animals. While there is a wide distinction in the dignity of the object of study, yet from a scientific point of view this separation is of little account. Pathological investigations of diseases of animals constitute no less genuine and valuable contributions to pathology in general than do similar investigations of human disease. The advancement of recent years in the education and aims of those who devote themselves to animal pathology, will serve to bring into closer relations the students of human and those of comparative medicine.

In the first place there are many diseases which are common to man and to animals. These can often be studied to greater advantage upon animals in which many conditions can be controlled which are beyond our control in man. In animals every stage of development of the disease can be studied, and in general, fresher material can be obtained. We can modify in various ways external and internal conditions so as to reach a clearer comprehension of the morbid processes. Moreover, the same disease may present interesting pathological peculiarities in different species of animals, so that the study of its occurrence in a single species affords incomplete knowledge. For instance, the pathologist whose sole knowledge of such a disease as tuberculosis is derived from the study of the disease as it occurs in man, has a far less complete understanding of this affection than one who is also familiar with the striking peculiarities of this affection in cattle, swine, fowls, and other animals.

Especial importance attaches, of course, to the study of such diseases as are communicable from animals to man, as, for instance, anthrax, glanders, tuberculosis, many entozoic affections, etc., and in general these are the animal diseases which have received the most attention from the students of human pathology.

One of the most important departments of comparative pathology is experimental pathology, the value of which to human pathology has long been recognized. To make of experimental pathology a distinct specialty and to endow it with a separate professorship, as is done in some foreign universities, does not seem to me to be in the direction of the most fruitful and healthy development. The experimental method is the handmaid of pathology in all its branches, and is the only means of solving many important problems. The experimental production of diseases in the lower animals affords an insight to be gained in no other way as to the causes, development, lesions and functional manifestations of many diseases. Experience, however, has shown that grave errors are likely to be committed by experimental pathologists who have no knowledge of the natural diseases and conditions of the animals used for experiments. How often, for example, have those studying the question of experimental tuberculosis mistaken for genuine tubercles nodules produced by parasitic entozoa, and to what misleading conclusions have such incorrect observations led.

There are many general pathological processes which can be studied to better advantage in animals than in man. Such subjects as inflammation, œdema, thrombosis, embolism, and infection have been elucidated in large part by observations made on animals. Due caution is, of course, to be exercised in applying such observations directly to human beings. Inasmuch as it is rarely possible for us to produce artificially all of the conditions which cause natural diseases, and as our very method of experimentation is in itself often a perturbing factor, it is no less important to study animal diseases resulting from natural causes, than it is to study the same diseases experimentally produced. Of course there are many diseases which have not yet been opened to the experimental method of investigation.

Questions of etiology and of pathogenesis are among those which have received, and are destined still further to receive, the greatest illumination from studies of comparative pathology. At present, probably no subject engages the attention of pathologists to a greater degree than the microscopic organisms which cause infection. If we had been confined to human beings in the study of infectious diseases, our knowledge in this direction would have been only a small fraction of what it is at present. In scarcely a single instance could the complete chain of proof required to demonstrate the causation of an infectious disease by a specific micro-organism have been furnished. The far-reaching principle of preventive vaccination or inoculation would not be known.

A most important and promising field of pathological study, at present only partly cultivated, is found in the infectious diseases of animals and of plants, not only on account of the great economic interests often involved, but also as a means of widening and deepening our conceptions as to the causes, development, prevention and treatment of infectious diseases in general. Any pathologist who is at all familiar with the remarkable and peculiar conditions under which the so-called Texas cattle fever of the United States develops and spreads, will realize that the complete elucidation of all the etiological factors of this disease not only would contribute to the solution of a great economic question, but also would open fresh points of view in our conceptions of infectious agents and their properties. When we consider the many conditions which it is in our power to control in studying animal diseases, and above all the possibility of submitting to an experimental crucial test our conclusions, it is clear that the study of natural and artificial infections, as well as of many other diseases in animals, is calculated to advance in the highest degree the science of pathology. It is not a small thing that questions which were once considered to be wholly transcendental, as for instance the doctrine of immunity against infectious diseases, have been brought within the working domain of experimental pathology.

But let us take a broader view of comparative pathology than that which considers abnormalities only in man and in animals related to man in structure and function. I believe that many problems and facts in human pathology await for their complete elucidation the same application of the comparative method of study which has made of normal anatomy virtually a new science. What a barren mass of apparently unrelated facts is human anatomy when studied without reference to comparative anatomy and embryology? If knowledge is the understanding of the real nature of a thing, and how it came to be as it is, then there is no knowledge of human anatomy without the aid of comparative anatomy and embryology. How difficult and unmeaning is the old method of studying the anatomy of the human brain and how fascinating does the anatomy of this organ appear in the light of development!

The interesting studies of heredity, by Weissmann and others, pertain in part to pathology and also illustrate brilliantly the value of the comparative method of research.

The application of embryology to the explanation of congenital malformations is familiar and has long been an acquisition of human pathology. More recent is the endeavor to refer the origin of the genuine tumors to anomalies in fœtal development. It is probable that experimental and comparative pathology also will shed much light upon the still obscure question as to the origin of tumors.

A large mass of observed pathological facts we must now accept without adequate explanation. It is often the fundamental and common morbid processes which are most obscure. For many of these we may hope to find satisfactory explanation in the results which the comparative study of pathology will afford. At present nothing is to be gained by attempting to generalize from scanty and incomplete observations in comparative pathology.

We must first accumulate a storehouse of facts. We need investigators who shall study pathological conditions not in man alone, or in the higher animals alone, but also in the simpler forms of plant and animal life. Something has been done in this direction, more indeed than is generally utilized in human pathology, but much more remains to be done. Conditions and processes which are difficult to comprehend in animals of complex organization often become clear in organisms of simple structure. Our pathological concepts are now derived almost wholly from observations made upon highly complex forms of life. I believe it to be no illusion to anticipate in thought a time when all forms and kinds of living matter will be included in the domain of pathology, and when pathological laws will be derived from results of investigations which begin with unicellular organisms and which end with man. By the adoption of this comparative method of study, pathology will in reality acquire greater simplicity and deeper significance than it now possesses.

